# Novel pyrrolopyrimidine derivatives: design, synthesis, molecular docking, molecular simulations and biological evaluations as antioxidant and anti-inflammatory agents

**DOI:** 10.1080/14756366.2022.2090546

**Published:** 2022-06-27

**Authors:** Amira I. Sayed, Yara E. Mansour, Mohamed A. Ali, Omnia Aly, Zainab M. Khoder, Ahmed M. Said, Samar S. Fatahala, Rania H. Abd El-Hameed

**Affiliations:** a Pharmaceutical Organic Chemistry Department, Faculty of Pharmacy, Helwan University, Cairo, Egypt; b Biochemistry Department, Faculty of Agriculture, Cairo University, Cairo, Egypt; c Medical Biochemistry Department, National Research Centre, Dokki, Egypt; d Department of Chemistry, The State University of New York, Buffalo, NY, USA; e Athenex Inc., Buffalo, NY, USA

**Keywords:** Pyrrolopyrimidines, cytotoxicity, macrophages-RAW 267.4, DPPH, molecular docking

## Abstract

Current medical approaches to control the Covid-19 pandemic are either to directly target the SARS-CoV-2 *via* innovate a defined drug and a safe vaccine or indirectly target the medical complications of the virus. One of the indirect strategies for fighting this virus has been mainly dependent on using anti‐inflammatory drugs to control cytokines storm responsible for severe health complications. We revealed the discovery of novel fused pyrrolopyrimidine derivatives as promising antioxidant and anti-inflammatory agents. The newly synthesised compounds were evaluated for their in vitro anti-inflammatory activity using RAW264.7 cells after stimulation with lipopolysaccharides (LPS). The results revealed that **3a, 4b,** and **8e** were the most potent analogues. Molecular docking and simulations of these compounds against COX-2, TLR-2 and TLR-4 respectively was performed. The former results were in line with the biological data and proved that **3a, 4b** and **8e** have potential antioxidant and anti-inflammatory effects.

## Graphical Abstract

**Figure:**
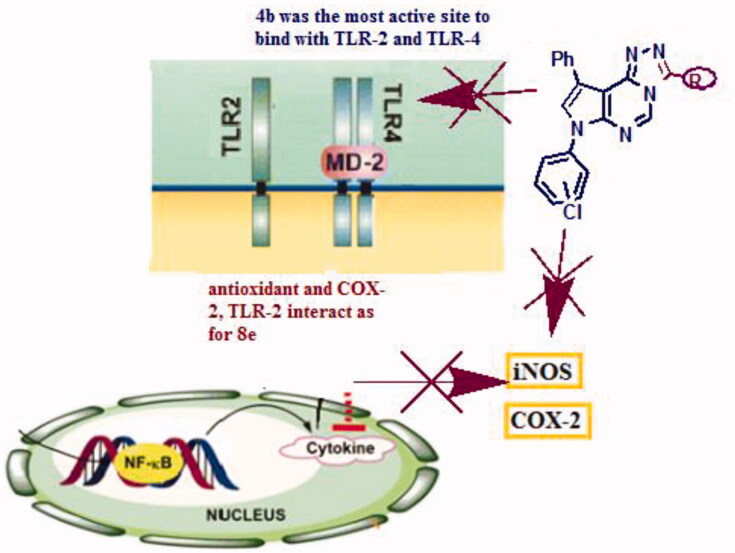

## Introduction

Toll-like receptors (TLRs) are a family of pattern recognition receptors (PRRs) that form the cornerstone of the innate sensor, and also shape and bridge innate and adaptive immune responses. They can recognise both the external pathogen-associated molecular patterns (PAMPs) and the internal damage-associated molecular patterns (DAMPs) are the most potent inducers of the inflammatory responses^1^^,^^2^. Recent studies emphasised that above 50% of the death toll worldwide is mainly due to chronic inflammatory diseases. TLR activation stimulates signalling cascades by the host as a defence mechanism against invaders and to repair the damaged tissue, leading to the release of various inflammatory cytokines and immune modulators^3^^,^^4^. However, excessive TLR activation disrupts the immune homeostasis by sustained pro-inflammatory cytokines and chemokine production and consequently contributes to the development and progression of many diseases, such as autoimmune diseases including lupus erythematosus and rheumatoid arthritis, cancer, sepsis, Alzheimer’s disease, and type 1 diabetes^5–11^.

TLRs serve as sensors of conserved components of microorganisms, such as, SARS-CoV-2, which triggers inflammatory signalling cascades, downstream transcription factors and induces the production of pro-inflammatory cytokines and over production of nitric oxide (NO) and reactive oxygen species (ROS)^12^. Cytokine storm is the major reason for the high mortality rates of COVID-19 due to the induction of excessive and prolonged high concentrations of pro-inflammatory cytokine/chemokine, besides the multiple organ dysfunction, which leads to physiological deterioration and death^13–15^. Reactive oxygen species (ROS) and reactive nitrogen species (RNS) are recognised for their dual role as both deleterious and beneficial species. Oxidative stress is the overproduction of ROS/RNS, viewed as an imbalance between the production of reactive species and their elimination by protective mechanisms, which leads to chronic inflammation and results in damage to cell structures, including lipids and membranes, proteins, and DNA, inhibiting their normal function^16^.

TLRs are classified into two subgroups such as cell membrane TLRs (TLR1, TLR2, TLR4, TLR5, TLR6, and TLR10) that are expressed on the cell surface and intracellular TLRs or nucleic acids sensors (TLR3, TLR7, TLR8, and TLR9) that are localised to the endoplasmic reticulum (ER), endosomes, and lysosomes^17–19^. The expression of these receptors not only on all innate immune cells such as macrophages, neutrophils, dendritic cells (DCs), basophils, natural killer (NK) cells, mast cells, and eosinophils but they are also present in a variety of cell types, including fibroblasts, endothelial cells, epithelial cells, and placental tissue. Moreover, the regulation for their locations are mainly in response to the recognised PAMP (recognizes invaders) and DAMPs (endogenous damage recognition)^20^^,^^21^. Structurally TLRs located on cell membranes possess an extracellular domain containing leucine-rich repeats that recognise distinct PAMPs and a toll-interleukin1 (IL-1) receptor (TIR) domain are required for downstream signalling. TLR4, the first toll protein homolog discovered in humans, was shown to induce the expression of genes involved in inflammatory responses. TLR2 and TLR4 have gained immense importance due to being among the cell surface TLRs^22^^,^^23^.

TLR4 is mainly activated by lipopolysaccharide (LPS), lipooligosaccharide (LOS), and lipid A from Gram-negative bacteria generally called endotoxin. The recognition through accessory molecules such as LPS-binding protein (LBP), the cluster of differentiation 14 (CD14), and myeloid differentiation factor 2 (MD2), but, heterodimerization of TLR2 with either TLR1or TLR6 is essential for recognising microbial cell wall component diacetylated and triacetylated lipopeptide^24^^,^^25^.

**Figure 1.**
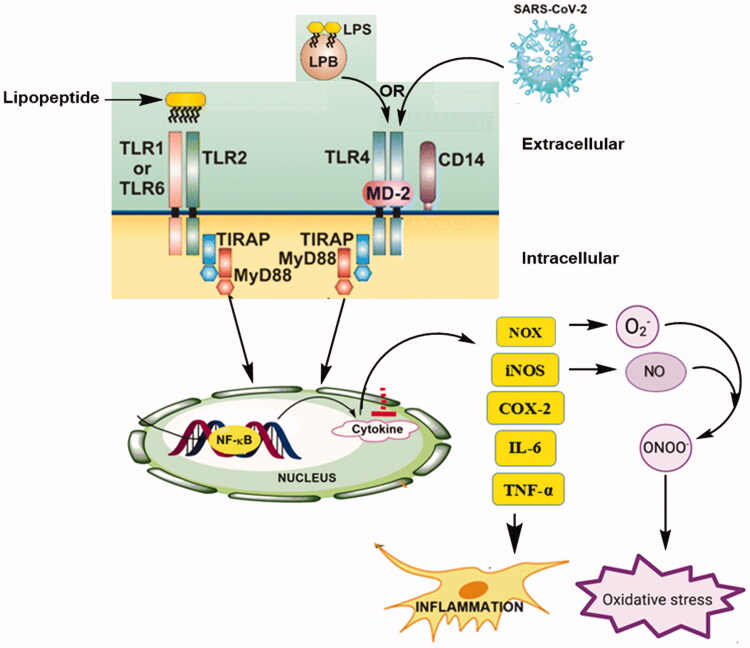
TLRs signalling activates transcription nuclear factor kB (NF-kB) in the nucleus and promotes the increase in the expression of the pro-oxidant enzymes NADPH-oxidase (NOX) and inducible nitric oxide synthase (iNOS), moreover pro-inflammatory cytokines tumour necrosis factor (TNF)-α, interleukin (IL)-1β, IL-6, and IL-12).

In response to TLR engagement, rapid induction of pro-inflammatory signalling starts with activation of the innate immune signalling cascade *via* both myeloid differentiation primary response protein 88 (MyD88)-dependent and MyD88-independent pathways. The MyD88-dependent signalling pathway is responsible for the early phase activation of transcription nuclear factor-kB (NF-kB) and mitogen-activated protein kinases (MAPKs); these events result in inducing the gene expression of pro-inflammatory cytokines [tumour necrosis factor (TNF)-α, interleukin (IL)-1β and IL-6], inflammatory mediators [reactive oxygen species (ROS), nitric oxide (NO), and prostaglandin E2 (PGE2)], which contribute to the progression of several inflammatory diseases^26–29^, as revealed in (Figure 1).

The activity of oxidant enzymes such as NADPH-oxidase (NOX), inducible NO synthase (iNOS) and cyclooxygenase-2 (COX-2); the enzymes involved in the production of ROS NO, and PGE2; is positively correlated with the expression of pro-inflammatory cytokines^30^^,^^31^. The suppression of inflammation through the discovery of a novel antagonists/inhibitors regulating TLR2/4 activity appears as a therapeutic strategy in the treatment of chronic inflammatory diseases^32^.

Inspired by the above-mentioned discoveries, and as continued for our effort in field for preparation of pyrroles as anti-inflammatory compounds^33–36^, some pyrrolopyrimidines **3–8** (namely, pyrrolotriazolopyrimidines and hydrazones derivatives) were synthesised, docked and screened for their antioxidant and anti-inflammatory activities *via* TLRs (TLR2 and TLR4) inhibition. Additionally, molecular dynamic simulations (MDS) were conducted for 100 ns using GROMACS 2.1.1 software using the docking coordinates of COX-2 and TLR-4 bound to compounds **4b** and **8e,** respectively. The MD simulation was performed to provide insights into precise estimation of the binding strength of a docked complex of COX-2 and TLR-4 bound to compounds **4b** and **8e**.

## Materials and methods

### Chemistry

#### Synthesis of lead compounds

All commercial chemicals used as starting materials and reagents in this study were purchased from Merck (Darmstadt, Germany) and were of reagent grade. All melting points were uncorrected and measured using Electro-thermal IA 9100 apparatus (Shimadzu, Japan); IR spectra were recorded as potassium bromide pellets on a Perkin-Elmer 1650 spectrophotometer (USA), Faculty of Science, Cairo University, Cairo, Egypt. ^1^H-NMR spectra were determined on a Varian Mercury (300 MHz) spectrometer (Varian UK) and chemical shifts were expressed as ppm against TMS as internal reference (The Main Chemical Warfare Laboratories, Almaza, Cairo, Egypt). Mass spectrum was carried out on Direct Inlet part to mass analyser on 70 eV (ISQ 7000, single quadrupole, GC-MS, Thermo Scientific, Massachusetts, USA) at the Regional Centre for Mycology and Biotechnology (RCMB), Al-Azhar University, Nasr City, Cairo, confirming the purity of the compounds as well as explore the characteristic fragmentation using EI mode and the expected [M. Wt]. Microanalyses were operated using Vario, Elmentar apparatus (Shimadzu, Japan), Organic Microanalysis Unit, Faculty of Science, Cairo University, Cairo, Egypt. Column Chromatography was performed on (Merck) Silica gel 60 (particle size 0.06–0.20 mm). All the listed compounds are new except compounds 1a,b were previously reported^37^^,^^38^.

### General procedure for the synthesis of compounds 2(a–b)

A mixture of 4-chloro pyrrolopyrimidine **1(a–b)** (0.01 mol), hydrazine hydrate (0.01 mol) was heated under reflux in absolute ethanol for 8 h, cooled, poured onto ice water to give precipitates which were filtered off, dried and recrystallized from methanol to give compounds **2(a–b)**.

#### (7–(3-Chlorophenyl)-5-phenyl-7H-pyrrolo[2,3-d]pyrimidin-4-yl)-hydrazine (2a)

Yield: 72%; m.p.: 204–206 °C; IR (KBr) υ (cm^−1^): 3413, 3320 (NH_2_), 3287 (N–H), 1533 (C=N); MS (EI) *m/z*: 337 (M + 2, 11.2%), 335 (M^+^, 34%), ^1^H NMR (DMSO-d_6_, 300 MHz) *δ* (ppm): 5.19 (br s, 2H, NH_2_, D_2_O exchangeable), 6.80–8.12 (m, 12H, Ar-H + NH, D_2_O exchangeable); Anal. Calcd for C_18_H_14_ClN_5_ (335.09): C, 64.48; H, 4.18; N, 20.90%. Found: C, 64.19; H, 4.12; N, 20.69%.

#### (7–(4-Chlorophenyl)-5-phenyl-7H-pyrrolo[2,3-d]pyrimidin-4-yl)-hydrazine (2b)

Yield: 80%; m.p.: 217–219 °C; IR (KBr) υ (cm^−1^): 3420, 3374 (NH_2_), 3233 (N–H), 1560 (C=N); MS (EI) *m/z*: 337 (M + 2, 18.7%), 335 (M^+^, 55.2%), ^1^H NMR (DMSO-d_6_, 300 MHz) *δ* (ppm): 5.26 (br s, 2H, NH_2_, D_2_O exchangeable), 6.78–8.09 (m, 12H, Ar-H + NH, D_2_O exchangeable); Anal. Calcd for C_18_H_14_ClN_5_ (335.09): C, 64.48; H, 4.18; N, 20.90%. Found: C, 64.22; H, 4.36; N, 20.71%.

### General procedure for the synthesis of compounds 3(a–b)

The appropriate hydrazine **2(a–b)** (0.01 mol) was heated under reflux for 8 h in formic acid (20 mL, 85%), cooled, poured onto ice water to give a precipitate which was filtered off, dried and recrystallized from ethanol to yield compounds **3(a–b)**.

#### 7–(3-Chlorophenyl)-9-phenyl-7H-pyrrolo[3,2-e][1,2,4]triazolo[4,3-c] pyrimidine (3a)

Yield: 77%; m.p.: 176–178 °C; IR (KBr) υ (cm^−1^): 1603 (C=N); MS (EI) *m/z*: 347 (M + 2, 8.92%), 345 (M^+^, 27.47%), ^1^H NMR (DMSO-d_6_, 300 MHz) *δ* (ppm): 6.51–7.87 (m, 11H, Ar-H), 8.24 (s, 1H, C5-H); ^13^C-NMR (DMSO, 75 MHz) *δ* (ppm): 95.8, 101.72, 105.36, 109.44, 111.3, 118.32, 119.27, 121.34, 122.65, 129.43, 129.88, 139.27, 144.45, 147.8, 156.3, 160.9, 162.3 (SP^2^ carbon atoms); Anal. Calcd for C_19_H_12_ClN_5_ (345.07): C, 65.09; H, 3.48; N, 20.29%. Found: C, 65.03; H, 3.42; N, 20.21%.

#### 7–(4-Chlorophenyl)-9-phenyl-7H-pyrrolo[3,2-e][1,2,4]triazolo[4,3-c] pyrimidine (3b)

Yield: 85%; m.p.: 190–192 °C; IR (KBr) υ (cm^−1^): 1614 (C=N); MS (EI) *m/z*: 349 (M + 2, 9.79%), 347 (M^+^, 29.14%), ^1^H NMR (DMSO-d_6_, 300 MHz) *δ* (ppm): 6.26–7.84 (m, 11H, Ar-H), 7.87 (s, 1H, C5-H); Anal. Calcd for C_19_H_12_ClN_5_ (345.07): C, 65.09; H, 3.48; N, 20.29%. Found: C, 65.25; H, 3.27; N, 20.24%.

### General procedure for the synthesis of compounds 4(a–b)

A mixture of the appropriate hydrazine **2(a–b)** (0.01 mol) and carbon disulphide (0.01 mol) was heated under reflux for 3 h in absolute ethanol (30 mL), cooled, poured onto ice water to give a precipitate which was filtered off, dried and recrystallized from ethanol to yield compounds **4(a–b).**

#### 7–(3-Chlorophenyl)-9-phenyl-7H-pyrrolo[3,2-e][1,2,4]triazolo[4,3-c]pyramid in-3-thione (4a)

Yield: 57%; m.p.: 193–195 °C; IR (KBr) υ (cm^−1^): 3322 (N–H), 1567 (C=N), 1487, 1258, 1020, 812 (C = S); MS (EI) *m/z*: 379 (M + 2, 11.06%), 377 (M^+^, 31.7%), ^1^H NMR (DMSO-d_6_, 300 MHz) *δ* (ppm): 6.93–8.30 (m, 11H, Ar-H), 10.23 (br s,1H, NH, D_2_O exchangeable); Anal. Calcd for C_19_H_12_ClN_5_S (377.06): C, 60.48; H, 3.18; N, 18.57%. Found: C, 60.45; H, 3.27; N, 18.73%.

#### 7–(4-Chlorophenyl)-9-phenyl-7H-pyrrolo[3,2-e][1,2,4]triazolo[4,3-c]pyramid in-3-thione (4b)

Yield: 60%; m.p.: 187–189 °C; IR (KBr) υ (cm^−1^): 3413 (N–H), 1609 (C=N), 1483, 1259, 1016, 800 (C = S); MS (EI) *m/z*: 379 (M + 2, 19.4%), 377 (M^+^, 58.6%), ^1^H NMR (DMSO-d_6_, 300 MHz) *δ* (ppm): 6.93–8.26 (m, 11H, Ar-H), 11.83 (br s,1H, NH, D_2_O exchangeable); Anal. Calcd for C_19_H_12_ClN_5_S (377.06): C, 60.48; H, 3.18; N, 18.57%. Found: C, 60.17; H, 3.11; N, 18.50%.

### General procedure for the synthesis of compounds 5(a–b)

The appropriate hydrazine **2(a–b)** (0.01 mol) was heated under reflux for 5 h in acetic anhydride (30 mL), cooled, poured onto ice water and neutralised with ammonia to give a precipitate which was filtered off, dried and recrystallized from ethanol to yield compounds **5(a–b)**.

#### 7–(3-Chlorophenyl)-3-methyl-9-phenyl-7H-pyrrolo[3,2-e][1,2,4]triazolo[4,3-c] pyrimidine (5a)

Yield: 59%; m.p.: 227–229 °C; IR (KBr) υ (cm^−1^): 1598 (C=N); MS (EI) *m/z*: 361 (M + 2, 15.7%), 359 (M^+^, 45.03%), ^1^H NMR (DMSO-d_6_, 300 MHz) *δ* (ppm): 2.10 (s, 3H, C3-CH_3_), 6.48–7.56 (m, 10H, Ar-H), 8.21 (s,1H, C5-H); ^13^C-NMR (DMSO, 75 MHz) *δ* (ppm): 39.56 (CH_3_), 99.7, 101.41, 104.33, 109.12, 112.33, 118.5, 121.48, 127.3, 129.43, 129.91, 137.66, 138.7, 139.2, 146.45, 152.49, 157.26, 161.7 (SP^2^ carbon atoms); Anal. Calcd for C_20_H_14_ClN_5_ (359.09): C, 66.85; H, 3.90; N, 19.50%. Found: C, 66.65; H, 3.74; N, 19.88%.

#### 7–(4-Chlorophenyl)-3-methyl-9-phenyl-7H-pyrrolo[3,2-e][1,2,4]triazolo[4,3-c] pyrimidine (5b)

Yield: 63%; m.p.: 201–203 °C; IR (KBr) υ (cm^−1^): 1572 (C=N); MS (EI) *m/z*: 361 (M + 2, 10.7%), 359 (M^+^, 30.33%), ^1^H NMR (DMSO-d_6_, 300 MHz) *δ* (ppm): 2.50 (s, 3H, C3-CH_3_), 6.51–7.56 (m, 10H, Ar-H), 8.61 (s,1H, C5-H); Anal. Calcd for C_20_H_14_ClN_5_ (359.09): C, 66.85; H, 3.90; N, 19.50%. Found: C, 66.88; H, 4.05; N, 19.64%.

### General procedure for the synthesis of compounds 6(a–b)

A solution of the appropriate hydrazine **2(a–b)** (0.01 mol) in pyridine (10 mL) was cooled in an ice bath, and an equimolar amount (0.01 mol) of ethyl chloroformate was added portion wise. Then the mixture was heated under reflux for 3 h, cooled, poured onto ice water and neutralise with HCl to give a precipitate which was filtered off, dried and recrystallized from ethanol to yield compounds **6(a-b).**

#### 7–(3-Chlorophenyl)-9-phenyl-7H-pyrrolo[3,2-e][1,2,4]triazolo[4,3-c]pyrimidin-3-one (6a)

Yield: 59%; m.p.: 226–228 °C; IR (KBr) υ (cm^−1^): 3402 (N–H), 1673 (C = O), 1512 (C=N); MS (EI) *m/z*: 361 (M + 2, 10.84%), 359 (M^+^, 29%), ^1^H NMR (DMSO-d_6_, 300 MHz) *δ* (ppm): 6.26–7.84 (m, 11H, Ar-H ), 7.87 (s, 1H, NH, D_2_O exchangeable); Anal. Calcd for C_19_H_12_ClN_5_O (359.07): C, 63.16; H, 3.32; N, 19.39%. Found: C, 63.31; H, 3.35; N, 19.35%.

#### 7–(4-Chlorophenyl)-9-phenyl-7H-pyrrolo[3,2-e][1,2,4]triazolo[4,3-c]pyrimidin-3-one (6b)

Yield: 63%; m.p.: 233–235 °C; IR (KBr) υ (cm^−1^): 3390 (N–H), 1681 (C = O), 1520 (C=N); MS (EI) *m/z*: 363 (M + 2, 8.87%), 361 (M^+^, 26.78%), ^1^H NMR (DMSO-d_6_, 300 MHz) *δ* (ppm): 6.90–7.87 (m, 11H, Ar-H ), 8.31 (s, 1H, NH, D_2_O exchangeable); ^13^C-NMR (DMSO, 75 MHz) *δ* (ppm): 98.04, 102.43, 109.14, 115.6, 116.49, 127.5, 129.20, 129.55, 130.48, 131.60, 133.42, 138.3, 157.41, 162.0 (SP^2^ carbon atoms), 166.91 (C = O), Anal. Calcd for C_19_H_12_ClN_5_O (361.07): C, 63.16; H, 3.32; N, 19.39%. Found: C, 63.15; H, 3.66; N, 19.02%.

### General procedure for the synthesis of compounds 7(a–b)

A mixture of the appropriate hydrazine **2(a–b)** (0.01 mol) and acetyl acetone (0.01 mol) in absolute ethanol was heated under reflux for 3 h, cooled, poured onto ice water to give a precipitate which was filtered off, dried, and recrystallized from ethanol to yield compounds **7(a–b)**.

#### 7–(3-Chlorophenyl)-4–(3,5-dimethyl-pyrazol-1-yl)-5-phenyl-pyrrolo[2,3-d]pyrimidine (7a)

Yield: 45%; m.p.: 180–182 °C; IR (KBr) υ (cm^−1^): 1608 (C=N); MS (EI) *m/z*: 401 (M + 2, 7.01%), 399 (M^+^, 20.68%), ^1^H NMR (DMSO-d_6_, 300 MHz) *δ* (ppm): 2.05 (s, 3H, CH_3_), 2.39 (s, 3H, CH_3_), 6.92–7.89 (m, 11H, Ar-H ), 8.32 (s, 1H, C-2 H); Anal. Calcd for C_23_H_18_ClN_5_ (399.12): C, 69.17; H, 4.51; N, 17.54%. Found: C, 69.20; H, 4.79; N, 17.35%.

#### 7–(4-Chlorophenyl)-4–(3,5-dimethyl-pyrazol-1-yl)-5-phenyl-pyrrolo[2,3-d]pyrimidine (7b)

Yield: 53%; m.p.: 199–201 °C; IR (KBr) υ (cm^−1^): 1617 (C=N); MS (EI) *m/z*: 401 (M + 2, 6.73%), 399 (M^+^, 19.3%), ^1^H NMR (DMSO-d_6_, 300 MHz) *δ* (ppm): 2.03 (s, 3H, CH_3_), 2.36 (s, 3H, CH_3_), 6.88–7.84 (m, 11H, Ar-H ), 8.31 (s, 1H, C-2 H); Anal. Calcd for C_23_H_18_ClN_5_ (399.12): C, 69.17; H, 4.51; N, 17.54%. Found: C, 69.36; H, 4.32; N, 17.66%.

### General procedure for the synthesis of compounds 8(a–f)

A mixture of the appropriate hydrazine **2(a–b)** (0.01 mol) and aromatic aldhyde (0.01 mol) was heated under reflux in absolute ethanol for 8 h, cooled, poured onto ice water to give precipitates which were filtered off, dried and recrystallized from ethanol to give compounds **8(a–f)**.

#### N-[(E)-benzylideneamino]-7–(3-chlorophenyl)-5-phenyl-pyrrolo[2,3-d]pyrimidin-4-amine (8a)

Yield: 46%; m.p.: 187–189 °C; IR (KBr) υ (cm^−1^): 3323(N–H), 1608 (C=N); MS (EI) *m/z*: 423 (M + 2, 20.45%), 421 (M^+^, 63.43%), ^1^H NMR (DMSO-d_6_, 300 MHz) *δ* (ppm): 5.43 (s, 1H, NH, D_2_O exchangeable), 6.43–7.94 (m, 15H, Ar-H), 8.26(s, 1H, C-2 H), 8.51 (s, 1H, CH).; Anal. Calcd for C_25_H_18_ClN_5_ (421.12): C, 70.92; H, 4.26; N, 16.55%. Found: C, 70.82; H, 4.51; N, 16.36%.

#### N-[(E)-benzylideneamino]-7–(4-chlorophenyl)-5-phenyl-pyrrolo[2,3-d]pyrimidin-4-amine (8b)

Yield: 60%; m.p.: 172–174 °C; IR (KBr) υ (cm^−1^): 3345(N–H), 1598 (C=N); MS (EI) *m/z*: 425 (M + 2, 7.01%), 423 (M^+^, 21.61%), ^1^H NMR (DMSO-d_6_, 300 MHz) *δ* (ppm): 5.21 (s, 1H, NH, D_2_O exchangeable), 6.79–7.53 (m, 16H, Ar-H), 7.95 (s, 1H, CH); Anal. Calcd for C_25_H_18_ClN_5_ (423.12): C, 70.92; H, 4.26; N, 16.55%. Found: C, 70.69; H, 4.20; N, 16.35%.

#### 7–(3-Chlorophenyl)-N-[(E)-(4-chlorophenyl)methyleneamino]-5-phenyl-pyrrolo[2,3-d]pyrimidin-4-amine (8c)

Yield: 65%; m.p.: 247–249 °C; IR (KBr) υ (cm^−1^): 3331(N–H), 1618 (C=N); MS (EI) *m/z*: 459 (M + 4, 3.3%), 457 (M + 2, 31.51%), 455 (M^+^, 47.38%), ^1^H NMR (DMSO-d_6_, 300 MHz) *δ* (ppm): 5.99 (s, 1H, NH, D_2_O exchangeable), 6.62–7.91 (m, 15H, Ar-H), 8.63 (s, 1H, CH); Anal. Calcd for C_25_H_17_Cl_2_N_5_ (455.08): C, 65.65; H, 3.72; N, 15.32%. Found: C, 65.80; H, 4.08; N, 15.37%.

#### 7–(4-Chlorophenyl)-N-[(E)-(4-chlorophenyl)methyleneamino]-5-phenyl-pyrrolo[2,3-d]pyrimidin-4-amine (8d)

Yield: 68%; m.p.: 187–189 °C; IR (KBr) υ (cm^−1^): 3338 (N–H), 1602 (C=N); MS (EI) *m/z*: 461 (M + 4, 2.61%), 459 (M + 2, 17.21%), 457 (M^+^, 25.61%), ^1^H NMR (DMSO-d_6_, 300 MHz) *δ* (ppm): 4.48 (s, 1H, NH, D_2_O exchangeable), 6.71–7.90 (m, 15H, Ar-H), 8.35 (s, 1H, CH); Anal. Calcd for C_25_H_17_Cl_2_N_5_ (457.08): C, 65.65; H, 3.72; N, 15.32%. Found: C, 65.55; H, 3.70; N, 15.36%.

#### 7–(3-Chlorophenyl)-N-[(E)-(4-methoxyphenyl)methyleneamino]-5-phenyl-pyrrolo[2,3-d]pyrimidin-4-amine (8e)

Yield: 72%; m.p.: 167–169 °C; IR (KBr) υ (cm^−1^): 3418 (N–H), 1604 (C=N); MS (EI) *m/z*: 455 (M + 2, 23%), 453 (M^+^, 67.39%), ^1^H NMR (DMSO-d_6_, 300 MHz) *δ* (ppm): 3.42 (s, 1H, OCH_3_), 5.26 (s, 1H, NH, D_2_O exchangeable), 6.71–7.66 (m, 15H, Ar-H), 7.88 (s, 1H, CH).; Anal. Calcd for C_26_H_20_ClN_5_O (453.13): C, 68.87; H, 4.42; N, 15.45%. Found: C, 68.87; H, 4.61; N, 15.44%.

#### 7–(4-Chlorophenyl)-N-[(E)-(4-methoxyphenyl)methyleneamino]-5-phenyl-pyrrolo[2,3-d]pyrimidin-4-amine (8f)

Yield: 80%; m.p.: 194–196 °C; IR (KBr) υ (cm^−1^): 3407 (N–H), 1605 (C=N); MS (EI) *m/z*: 455 (M + 2, 8.91%), 453 (M^+^, 25.35%), ^1^H NMR (DMSO-d_6_, 300 MHz) *δ* (ppm): 4.12 (s, 1H, OCH_3_), 4.50 (s, 1H, NH, D_2_O exchangeable), 6.89–8.18(m, 15H, Ar-H), 8.28 (s, 1H, CH).; Anal. Calcd for C_26_H_20_ClN_5_O (453.13): C, 68.87; H, 4.42; N, 15.45%. Found: C, 68.77; H, 4.34; N, 15.17%.

### Molecular docking

All compounds were constructed using MOE 2014.09 and filed in a molecular database file^39^. The crystal structure of COX-2 TLR-2 and TLR-4 were downloaded from the protein data bank (**PDBID: 4COX, 2Z80** and **2Z63**; respectively)^40–42^. Protein was energy diminished and 3 D protonated *via* the structure preparation module of MOE. The co-crystallized bound compound and water molecules were removed from the crystal structure. The site of docking was recognised and the database containing all the tested compounds has been established using rigid receptor as a docking protocol and triangle matcher as a placement method. Two rescoring functions were selected, London dG and GBVI/WSA dG. The force field was used as a refinement. Free binding energy (kcal/mol) was calculated, and only the best-scored pose was selected for each compound.

### Molecular dynamic simulation

Four molecular dynamic simulations (MDS) were conducted for 100 ns using GROMACS 2.1.1 software^43^. The retrieved docking coordinates of COX-2 and TLR-4 bound to **4b** and **8e** were used as input structures for the molecular dynamics. The receptor and ligand topologies were generated by PDB2gmx (embedded in GROMACS) and GlycoBioChem PRODRG2 Server respectively, both under GROMOS96 force field^44^. After rejoining ligands and receptor topologies to generate the four systems, the typical molecular dynamics scheme of GROMACS was applied for all the systems. This includes solvation, neutralisation, energy minimisation under GROMOS96 43a1 force field and two stages of equilibration (NVT and NPT)^45^. Finally, unrestricted production stage of 100 ns was applied for the four systems with particle mesh ewald (PME) method implemented to compute the long-range electrostatic values using 12 Å cut-off and 12 Å Fourier spacing. The stability of the complexes was judged using RMSD and RMSF values calculated from the MDS trajectories from the production step.

### MM-PBSA calculation and per residue contribution

The MM-PBSA package of Kumari et al.^46^ was contrived to calculate the binding free energy between the ligands and the two receptors using the following equation. All four complexes were subjected to such calculations.

$$\Delta {\text{G}}_{\left(\text{Binding}\right)}={\text{G}}_{\left(\text{Complex}\right)}-{\text{G}}_{\left(\text{Receptor}\right)}-{\text{G}}_{\left(\text{Ligand}\right)}$$

### Biology

#### Antioxidant activity of tested compounds using 2,2-Diphenyl-1-picrylhydrazyl (DPPH) radical scavenging protocol

DPPH scavenging potential of samples was determined with a slightly modified method^47^^,^^48^. Each sample was prepared at 500 ppm. Serial concentrations of samples were prepared (200 µL, 400 µL and 800 µL). Methanol was added to complete the total volume to 1 mL and all the samples were vortexed well. 1 mL of 0.1 mM DPPH methanolic solution was added to each diluted sample. All the samples were vortexed again, then left to stand for 30 min in the dark at environment temperature. The absorption of the developed colour appeared against the blank reagent was measured at 517 nm using a spectrophotometer. BHT was used as a standard antioxidant. The capability to scavenge DPPH radical was estimated using the following equation:

DPPH scavenging ability (% inhibition)=[(A0−A*)/A0]×100

A0 is Control absorption and A* is sample absorption.

### Anti-inflammatory activity

#### Cell culture (seeding and treatment)

The macrophage cell line, RAW 264.7 was obtained from the ATCC (American type culture collection). The cells were cultured in RPMI 1640 medium (Roswell Park Memorial Institute) and supplemented with 1% pen/strep and 10% heat-inactivated foetal bovine serum. The cells were incubated, in a humidified incubator, in an atmosphere of 5% CO_2_ at 37 °C and were subculture twice before the experiment^49^.

RAW 264.7 cells were suspended in a RPMI medium. After 24 h of seeding 1 × 105 cells per well (in 96 well plates) and incubated for 24 h for the experiments. The cells were then treated with the samples at concentrations of 100, 50, 25 and 12.5 μg/mL and incubated for 1 h. They were then stimulated with 10 μg/mL of LPS for another 24 h. The supernatant was gently transferred to new 96-well plates and used for NO determination, while the cells that remained in the old plate were used for the MTT assay of cell viability. Samples (stock) were dissolved in DMSO, and the working samples were prepared in the media. Cell viability was assessed by the mitochondrial dependent reduction of yellow MTT (3–(4,5-dimethylthiazol-2-yl)-2,5-diphenyltetrazolium bromide) to purple formazan ^49^.

The percentage of change in viability was calculated according to the formula:

$$[(\frac{\text{Reading of extract }}{\text{Reading of negative control }})-1]\times 100.$$

### Nitric oxide assay

Nitric oxide production was assayed by measuring nitrite in the supernatants of cultured LPS-RAW 264.7 cells. The assay was carried out as described previously with slight modification^48^^,^^50^. After pre-incubation of RAW 264.7 cells (1 × 105 cells/mL) with LPS (10 µg/mL) for 24 h, the amount of nitrite, a stable metabolite of NO used as an indicator of NO production in the culture medium was measured using the Griess reagent (1% sulphanilamide and 0.1% naphthyl ethylenediamine dihydrochloride in 2.5% phosphoric acid). A volume of 50 µL of the cell culture medium was mixed with 50 µL of the Griess reagent. Subsequently, the mixture was incubated at room temperature for 15 min and the absorbance was measured at 540 nm by a microplate reader. Fresh culture medium was used as a blank in every experiment. The quantity of nitrite was determined from a sodium nitrite standard curve as expressed in the equation.

$$\text{Nitric oxide inhibition }(\%)=(\frac{\text{optical denisty of control}-\text{optical denisty of test }}{\text{optical denisty of control }})\;\times 100.$$

## Results

### Chemical results

**Scheme 1.**
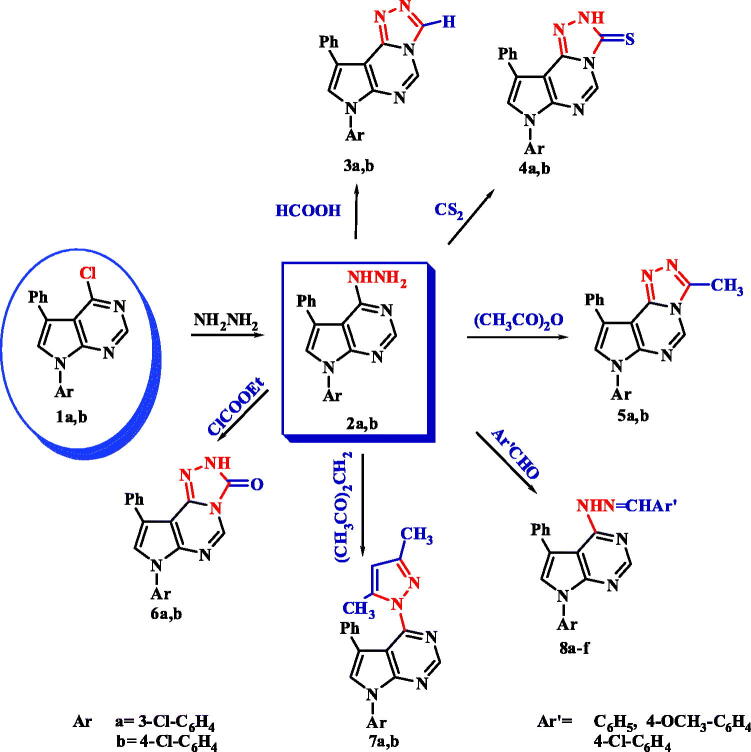
Synthesis of Pyrrolopyrimidines and Pyrrolotriazolopyrimidines (**2–8**).

The remarkable biological activity of pyrrolopyrimidines and fused pyrrolopyrimidine derivatives^51–54^ has inspired us to synthesise new derivatives and test their anti-inflammatory activity^55^^,^^56^. The synthetic strategies for our target compounds are presented in Scheme 1.

The previously reported 4-chloropyrrolopyrimidine derivatives **1(a–b)** were heated under reflux, independently, with hydrazine hydrate in absolute ethanol to afford 4-hydrazino-pyrrolopyrimidines **2(a–b)**, which were subsequently used as starting materials for the other novel derivatives^38^^,^^57^. In brief, pyrrolo[3,2-*e*][1,2,4]triazolo[4,3-*c*]pyrimidine derivatives **3(a–b)** and **6(a–b)** were obtained *via* reaction of hydrazino derivatives **2(a–b)** with formic acid, CS_2_, acetic anhydride and ethyl chloroformate, respectively^52^^,^^58^. Analysis of the spectral data of the new compounds confirm their structure in many features; as the disappearance of NH_2_ group absorption bands in IR spectra as well as its signal in ^1^H-NMR spectra, also that of NH group in some of these compounds, increasing number of aromatic protons, the appearance of amide or thioamide distinctive peaks. Additionally, 4-Hydrazino derivatives **2(a–b)** were also reacted with acetylacetone to produce 4-pyrazolyl-pyrrolopyrimidines **7(a–b).** Finally, *N*-(arylidineamino)pyrrolopyrimidine-4-amines **8(a–f)** were obtained from the condensation reaction of **2(a–b)** with different aromatic aldehydes. The structures of all the produced compounds were supported with elemental analysis and spectral data^51^^,^^53^.

#### Biological evaluation

In this study, we examined the effect of 18 newly synthesised compounds against reactive oxygen species (**ROS**), using 1,1-diphenyl-2-picrylhydrazyl (**DPPH**) radical scavenging assay using a well-known antioxidant control drug butylated hydroxytoluene (**BHT**). BHT is a lipophilic organic compound frequently used antioxidant recognised as safe for use in foods, pharmaceuticals and different industries^49^^,^^59^. Three compounds (namely **triazolo-pyrrolopyrimidines 3a, 4b,** and **arylidineaminopyrrolopyrimidine 8e)** showed significant activity against the standard reference (**BHT**).

The DPPH assay is the commonly used assay to define the promising antioxidant compounds, which act as free radical scavengers *in vitro*^60^. All the tested compounds exhibited poor or no scavenging properties against the DPPH radical, except for three compounds (**3a, 4b,** and **8e)** and the %inhibition was proportional to the concentration of each compound. Compounds **3a, 4b,** and **8e** showed promising anti-oxidative activities compared to the reference, **BHT**. Compound **8e** revealed the premier DPPH-scavenging activity, followed by compounds **4b** and **3a** as shown in Table 1.

**Table 1.** DPPH radical-scavenging activity of active compounds^a^ against reference anti-oxidant BHT.

Conc.	Active comp.			
	% Inhibition (mean ± SEM)			
	100	200	400	IC50 µg/mL
**3a**	39.56 ± 0.88	58.09 ± 1.50	62.51 ± 0.32	160.05
**4b**	37.97 ± 0.71	77.76 ± 1.60	79.55 ± 1.06	129.38
**8e**	40.63 ± 2.13	82.15 ± 0.14	85.22 ± 0.17	122.07
**BHT**	40.03 ± 0.39	76.81 ± 0.21	90.07 ± 0.26	128.77

^a^All compounds (**3–8**) were tested against DPPH; result represent the most active compounds.

According to the data in Table 1, the ideal radical-scavenging activity of our samples was demonstrated by compound **8e** at concentration 200 µg/mL which provides the highest antioxidant activity when compared to the same concentration of **BHT**. Compound **4b** also exhibited significant activity (with IC_50_ ≈129 µg/mL), which is similar to that of BHT followed by compound **3a** (IC_50_ ≈160 µg/mL).

The determination of the cytotoxic effect of our active antioxidant compounds on normal macrophages was critical, as shown in previous studies, many bioactive compounds were reported as toxic agents to normal cells and could be responsible for cells death by disrupting protein synthesis^48–50^^,^^61^. Table 2 shows the possible cytotoxic activity of our active compounds against macrophage cell line RAW 264.7.

**Table 2.** Cytotoxicity of compounds **3a, 4b** and **8e** against RAW macrophage cells.

Active comp.	Cytotoxicity of raw cells% (mean ± SEM)			
	100 µg/mL	50 µg/mL	25 µg/mL	12.5 µg/mL
**3a**	92.0 ± 1.6	78.7 ± 2.2	76.5 ± 1.9	0
**4b**	85.4 ± 0.6	75.2 ± 2.5	69.6 ± 1.3	0
**8e**	77.7 ± 1.5	73.1 ± 1.3	65 ± 3.1	0
LPS (−ve control)	–	–	–	–

As shown in Table 2, the highest cytotoxic activity was observed by compound **3a** at a concentration of 100 µg/mL and the lowest cytotoxic activity was observed by compound **8e** at a concentration of 25 µg/mL. The cytotoxic activity of our compounds could be attributed to a variety of factors, including the induction of cell damage, the initiation of various immune system reactions, and the electrostatic attraction of sample, and treated cells. These findings suggest that their cytotoxicity was most likely caused by low glutathione levels, high lipid peroxidation, and reactive oxygen species in responsive genes, which caused DNA damage and necrosis, followed by the evaluation of nitric oxide (NO) production and LPS-induced cytotoxicity and inflammatory response in RAW 264.7

The effect of different concentrations of our tested compounds on nitric oxide (NO) was investigated using the Griess assay to estimate nitrite accumulation in the cultivating medium^48^^,^^62–66^.

The data presented in Table 3 demonstrated that our compounds significantly inhibited LPS- stimulated NO production by LPS- induced RAW 264.7 macrophages. The highest inhibition activity was observed by compound **8e** at IC_50_ ≈ 53 µg/mL

**Table 3.** Anti-inflammatory activity of compounds **3a, 4b** and **8e** against nitric oxide.

Conc.	Comp.				
	NO % inhibition (mean ± SEM)				IC_50_ µg/mL
	100 µg/mL	50 µg/mL	25 µg/mL	12.5 µg/mL	
**3a**	65.6 ± 1.2	62.5 ± 1.2	53.1 ± 1.3	40.6 ± 2.6	57.3
**4b**	58.1 ± 3.5	54.3 ± 1.6	52.8 ± 1.1	39.8 ± 1.9	64.8
**8e**	71.8 ± 1.0	67.5 ± 1.8	66.5 ± 3.7	38.7 ± 1.7	52.5

## Discussion

Inflammation and oxidative stress are considered exceptionally related events; both are considered the main factor in many chronic diseases as lung injury and COVID-19. Moreover, increasing evidence shows that oxidative stress is recognised as the key path-way affecting the severity of lung injury^13^^,^^67–69^. Macrophages play critical roles in the initiation of inflammatory responses through secretion of a great number of pro-inflammatory mediators and cytokines, including tumour necrosis factor-α (TNF-α), interleukin-1β (IL-1β), IL-6, inducible nitric oxide synthase (iN-OS) and cyclooxygenase-2 (COX-2)^14^^,^^70–72^. In early responses to inflammation, specific damage-associated molecular patterns are recognised by immune cell pattern recognition receptors (PRRs), including toll-like receptors (TLRs), pattern recognition receptors (PRR) recognise pathogen-associated molecular patterns (PAMPs)^73–75^. TLR-4 can be also activated by damage-associated molecular patterns (DAMPs) and endogenous agonists released by injured tissues and necrotic cells^76–82^. TLR4-mediated inflammation, triggered by DAMPs, is involved in several diseases such as sepsis. Sepsis is one of the potential medical complications in severe influenza and recently in SAR-CoV-2 infection; leading to life-threatening organ dysfunction^7^.

Septic shock with overexpression of pro-inflammatory cytokines could be competently blocked by using TLR4 antagonists. Selective TLR4 antagonists as Eritoran (E5564) and TAK-242 were first progressed to clinical trials for the treatment of sepsis and have been discontinued in different phases. Yet, due to COVID −19 pandemic crisis, Eisai Co., Ltd. is participating in the global network REMAP-CAP-COVID (Randomized, Embedded, Multi-factorial, Adaptive Platform-Community Acquired Pneumonia COVID), which aims at developing therapeutics drugs for the novel coronavirus through drug repurposing, hoping to control the cytokine storm and prevent pneumonia complications through inhibiting the activation of TLR4 located at the uppermost stream of various cytokine production signals that cause cytokine storms, involved in the aggravation of pneumonia caused by coronavirus^67^.

**Figure 2.**
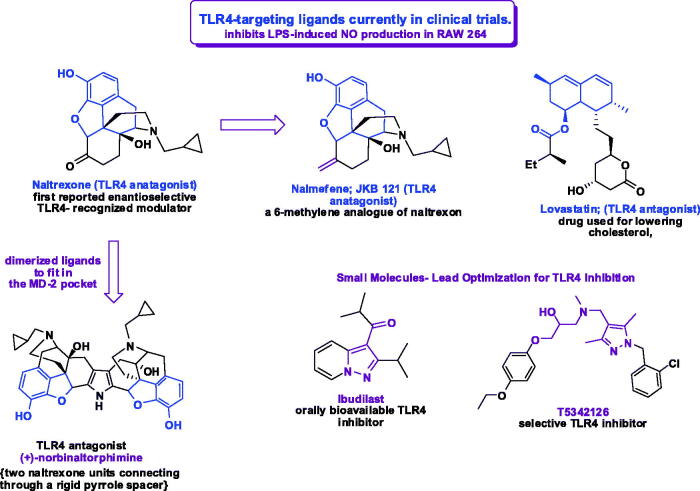
TLR4-targeting ligands currently in clinical trials.

Based on the nature of LPS, a hydrophobic lipid A domain, as TLRs agonist; several synthetic compounds have been developed as adjuvants for clinical use (namely; small-molecule inhibitors, peptides, microRNAs, nanoparticles, lipid A analogs, and derivatives of natural products)^79^^,^^80^^,^^83–86^. Recently, a bivalent ligands containing pharmacophores derived from naltrexone has reported showing a high selectivity. The MD-2 binding pocket is much larger than the size of (+)-naltrexone, which allows the discovery of ligand dimerisation of two bivalent ligands by connecting two naltrexone units through a rigid pyrrole spacer. Screening of clinically approved drugs with high blood − brain barrier permeability was also considered due to the high failure rate of new TLR-4 antagonist. Lovastatin, a well-known anti-hyperlipidemic drug, was recognised as a specific TLR4 antagonist, small-molecule acting as TLR-4 antagonist, can either block ligand-receptor interaction or cause dimerisation of the TLR4-MD2 complex. As an example; TAK-242 (resatorvid), a cyclohexane derivative that binds to the SH_2_ group of cysteine of the TIR domain. A previously reported compound T5342126 (Figure 2), as a selective TLR4 inhibitor was found to be an inhibitor of the interaction interface of TLR4-MD2^61^^,^^87–91^. Molecular docking was performed and revealed that the benzyl group of this compound binds with the hydrophobic pocket of TLR4, and the carbazole group occupies the MD2 pocket.

New approaches to the relation between TLRs and COX-2 also investigated several inflammatory pathways including inflammatory arthritis, cancer and diabetic nephropathy^92–96^. Experiments using TLR2 deficient, TLR4-deficient, and NLRP3-deficient mice indicated that these three proteins are involved in macrophage prostaglandin E2 (PGE2) secretion. Also, it has been reported that the immune responses to induced brain abscesses did not only depend on TLR2 but also TLR4 was required, suggesting that they play key roles in immune response modulation, inflammation, and induced PGE2; an endogenous lipid mediator that is essential for pathological conditions, immune cell secretion of different organs and also act as an inflammatory mediator. Sign for response to the acute inflammatory stimulation, are the release of prostaglandins (PG), and leukotrienes. In which the inhibition of PGE synthesis is considered an important anti-inflammatory strategy^93^^,^^97^^,^^98^. PGE2 is generated by the conversion of arachidonic acid (AA), which is released and used by 2 different cyclooxygenases (COXs), COX-1 (constitutive) and COX-2 (inducible)^53^^,^^74^^,^^88^^,^^89^^,^^91^^,^^99–104^. COX-2 is responsible for prostaglandin production during different pathological processes involving inflammation^97^^,^^98^^,^^105^. Recently, it is assumed that inflammation can lead to carcinogenesis by generation of ROS that can damage DNA, and excessive production of cytokines, which will regulate the COX-2/PGE2 (prostaglandin E2) signal pathway in inflammation and cancer cells^93^^,^^97^^,^^98^^,^^106^. Providing new evidence through the inhibition of inflammatory cytokine expression, which can aid in dimensih tumour development and progression. NSAIDs are investigated for the prevention of cancer progression and metastasis, particularly in the case of colon cancer^97^^,^^98^.

**Figure 3.**
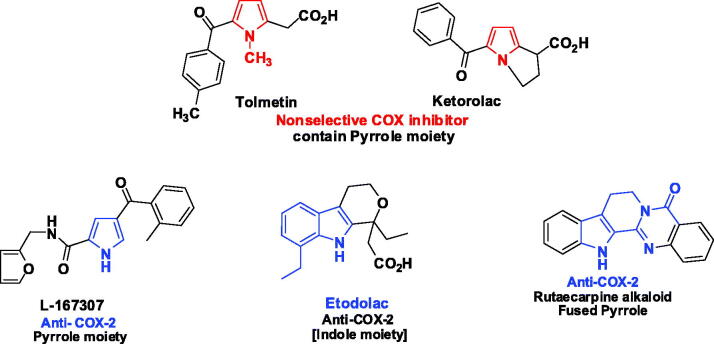
Pyrrole and fused pyrrole as selective COX-2 inhibitor^31^.

Many pathways have been reported for the LPS-macrophages activated by the TLR4 agonist and/or induced by TLR2/MyD88 activation, which is important for inflammatory processes are characterised by increased COX-2 accounting for the bulk of PGE biosynthesis^93^^,^^107^. NSAIDs inhibit both COXs enzymes and decrease production of PGE2 among these pyrrole containing compounds^108–110^. These compounds block PGE synthesis by non selective inhibition (indomethacin, acemetacin, tolmetin and ketorolac) or by selective-inhibition of COX-2 (etodolac). Although the overmentioned importance, lately the concern about drug interactions between widespread NSAIDs and cardiovascular treatments has been provided in several clinical settings^111^^,^^112^. The cardiovascular safety of NSAIDs have arisen initially because of reported associations between rofecoxib (COX-2 selective inhibitor) and myocardial infarction, causing rofecoxib to withdrawn from market in 2004^113^. Others COX-2-selective inhibitors, namely; celecoxib, valdecoxib, parecoxib, etoricoxib and lumiracoxib. All have shown a reduced risk of inducing gastroduodenal injury. Cardiovascular risk was also reported in for celecoxib, yet the evidence showed that the cardiovascular risks of celecoxib is less than that with rofecoxib, appeared to be dependent on the individual drugs. New compounds have been added to NSAIDS a day on to overcome the mention side effects among theses and in order drug discovery of new anti-inflammatory drugs with high safety targeting the TLRs as new potent anti-inflammatory novel compounds.; among these a series of *N*-pyrrolylcarboxylic acids have been reported as potent COX-2 inhibitors^114^^,^^115^, as revealed in (Figure 3).

**Figure 4.**
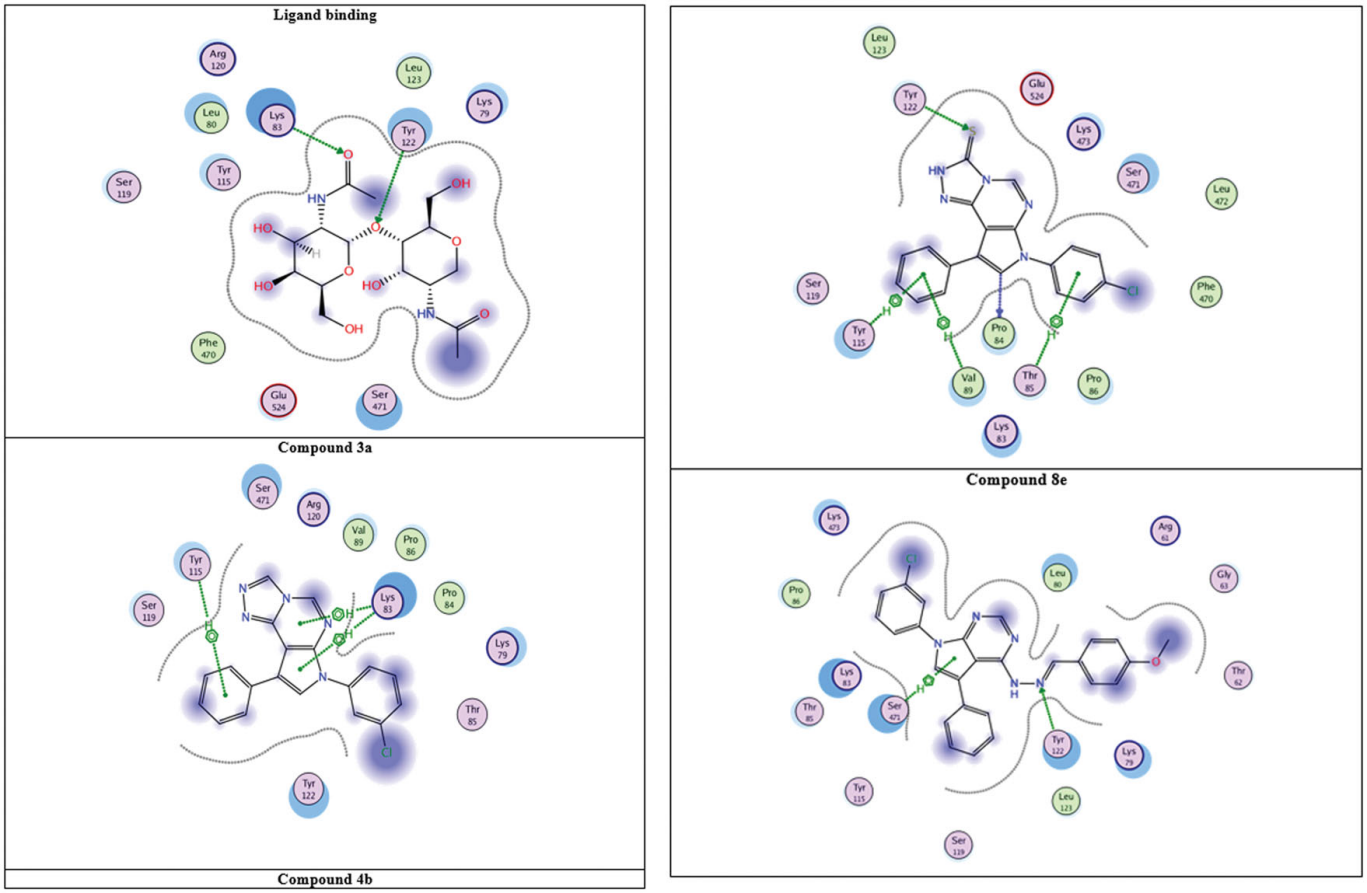
The binding modes of the active compounds with COX-2 active site.

**Figure 5.**
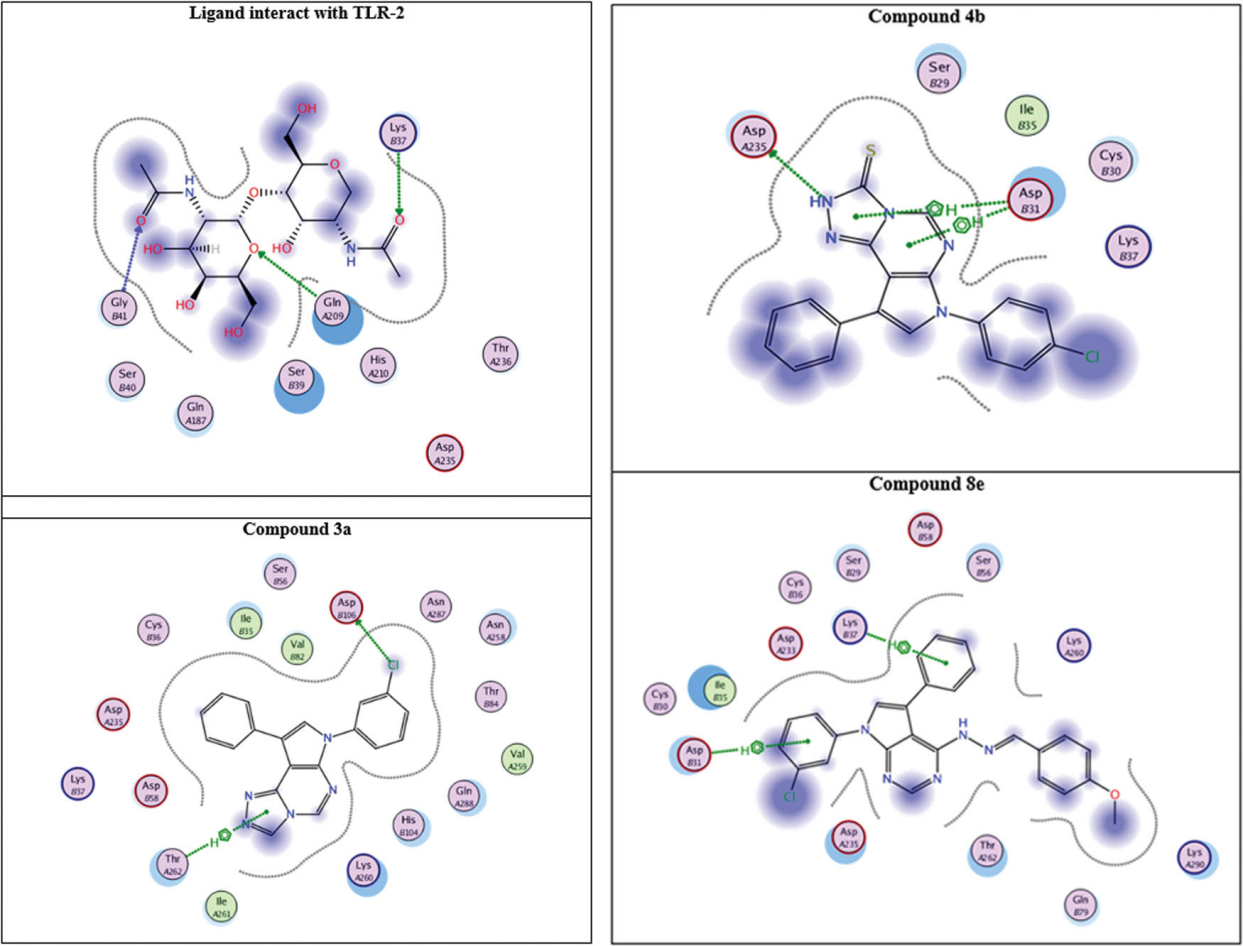
Interact with TLR-2 active site.

**Figure 6.**
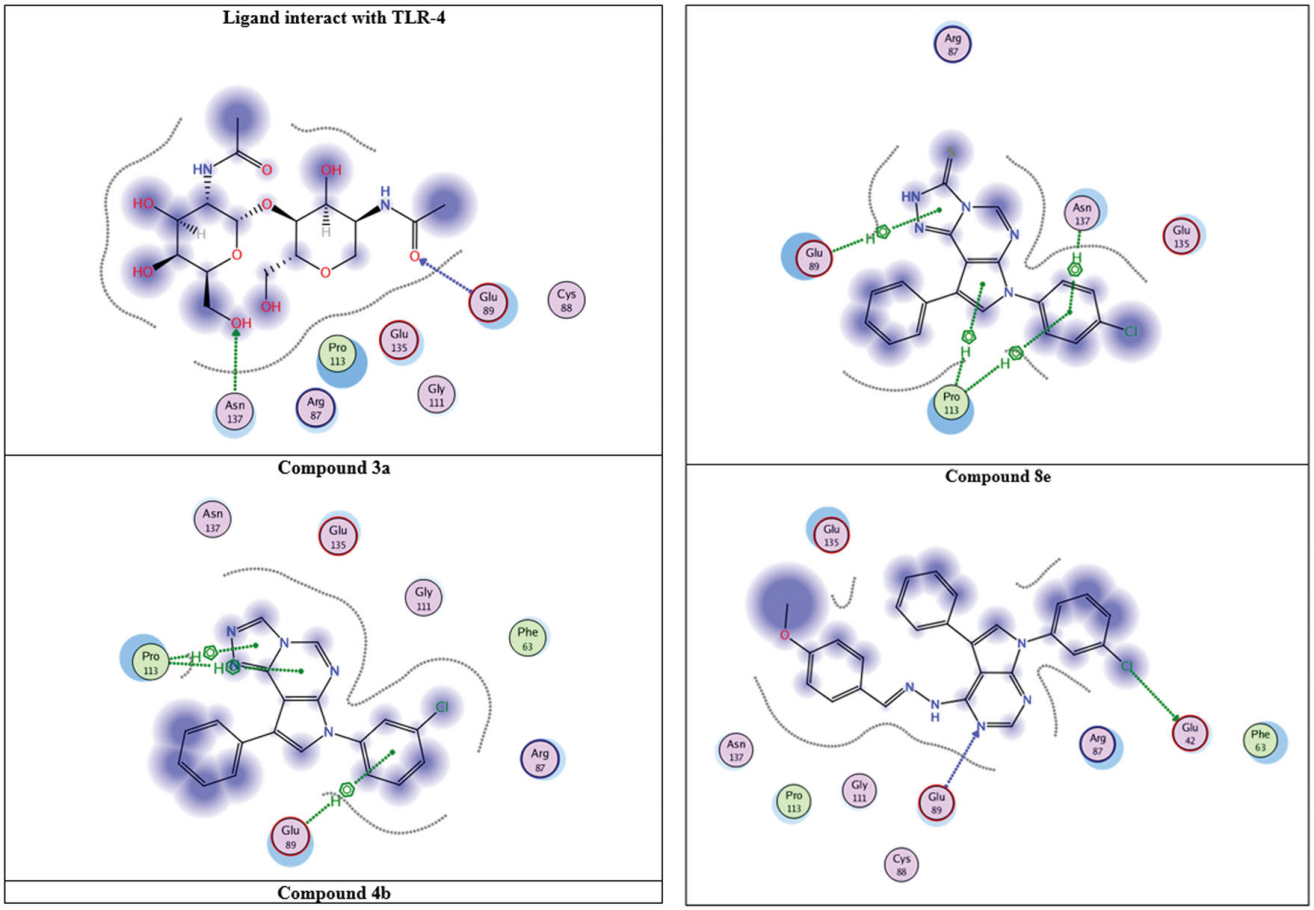
Interact with TLR-4 active site.

To understand the biological results presented herein of our active compounds, a molecular docking study was performed using the MOE 2014.09 software. Molecular docking screenings were performed after achieving synthesis and characterisation of the all-new compounds. The potential binding modes for the most active compounds (**3a, 4b**, and **8e**) was explored within the active site of the COX-2; both TLR-2 and TLR-4 were used. The binding affinity of the highly active compounds was calculated inside all the three enzymes’ binding sites^73^^,^^83^^,^^89^ as shown in Figures 4–6.

The binding modes of the active compounds inside the active site of COX-2 were evaluated using the protein coordinates (PDB 4COX) of COX-2 inbound with its ligand (Indomethacin). All the newly synthesised compounds were docked into the active site of COX-2. The results confirmed that the three compounds: triazolopyrrolopyrimidines **3a**, **4b**, and arylidineaminopyrrolo-pyrimidine **8e** had the lowest clash score, which confirm their well-fitting in the binding site and resulted in the highest affinity values within MOE 2014.09 docking results as revealed in Figure 4.

As shown in Figure 4 and Table 4, the reference ligand forms hydrogen bonding with both Tyr 122 and Lys 83 amino acid residues as the main non-covalent interactions with the protein. Compound **4b** showed the highest binding affinity, forming an H-bond with both of the Tyr 122 and Pro 84 amino acid residues. Furthermore, three more hydrophobic interactions were recognised with Tyr 115, Val 89 and Thr 85 amino acid residues. Compound **8e** shows good binding affinity resulting from both hydrophobic interaction with Ser 471 and hydrophilic hydrogen bonding (using hydrazine N) with Tyr 122 amino acid residue. For compound **3a**, two hydrophobic interactions were observed with Lys 83 and Tyr 115 amino acid residues. The *in silico* results of compounds **3a, 4b** and **8a** were promising and a further investigation of three compounds using DPHH and NO assay was performed as previously mentioned. The three compounds showed promising *in-vitro* activities as well. To understand the complete mechanism of these compounds further docking experiments were performed on both TLR-2 (PDB; 2Z80) and TLR-4(PDB; 2Z63), as revealed in (Figures 5 and 6).

**Table 4.** Results for molecular docking studies of compounds **3a, 4b** and **8e** versus reference in COX-2 active site (PDB: 4COX).

Compound	Docking score (s) Kcal/mol	RMSD	E score 1 (London dG) Kcal/mol	E score 2 (London dG) Kcal/mol	Binding interaction (ligand-receptor)
**3a**	−4.8144	1.9552	−8.0943	−4.8144	(Pyrimidine-LYS83) (pi-H, 3.54 Å) (Pyrrole-LYS83) (pi-H, 3.87 Å) (Benzene-TYR115) (pi-H, 3.70 Å)
**4b**	−5.2967	1.3831	−8.4435	−5.681	(Pyrrole C-PRO84) (H-b, 2.82 Å) (S-TYR122) (H-b, 3.09 Å) (7-Benzene-THR85) (pi-H, 3.91 Å) (9-Benzene-VAL89) (pi-H, 3.95 Å) (9-Benzene-TYR115) (pi-H, 3.50 Å)
**8e**	−5.4527	1.5696	−8.6607	−5.4527	(N arylidine-TYR122) (H-b, 3.00 Å) (Pyrrole-SER471) (pi-H, 3.66 Å)
Ligand (indomethacin)	−4.3598	2.4889	−9.9341	−4.3598	(O-TYR122) (H-b, 2.88 Å) (O-LYS83) (H-b, 2.83 Å)

As shown in Figure 6 and Table 5, the three active compounds (**4b**, **3a** and **8e**) were found to occupy the same binding site as the reference ligand. For the reference ligand, all non-covalent interactions formed are mostly hydrophilic i.e. COOH and O atom of the ligand interact with Lys B37, Gly B41 and Gln A209, forming Hydrogen bonding with its oxygen. Compound **4b** showed the highest binding affinity to the receptor by forming hydrophobic and hydrophilic interactions (three binding poses). These interactions are as follows: Two hydrophobic interactions with Asp B31 and one hydrogen bonding with Asp A235. Similar to what was shown for COX-2, compound **8e** is still showing the second-best receptor interactions with two hydrophobic interactions with Lys B37 and Asp B31. Furthermore, compound **3a** showed very good binding affinity with both hydrophobic interactions with Thr A262 and hydrophilic interaction between the − Cl and Asp B106, supporting its good activities in NO-assay (Table 6).

**Table 5.** Results for molecular docking studies of compounds **3a, 4b** and **8e** versus reference in TLR-2 active site (PDB: 2Z80).

Compound	Docking score (s) Kcal/mol	RMSD	E score 1 (London dG) Kcal/mol	E score 2 (London dG) Kcal/mol	Binding interaction (ligand-receptor)
**3a**	−5.1732	1.2973	−7.7491	−4.5062	(Cl-ASPB106) (H-b, 3.14 Å) (Triazole -THRA262) (pi-H, 3.82 Å)
**4b**	−4.1056	0.9909	−7.353	−4.1056	(N-ASPA235) (H-b, 3.18 Å) (Pyrimidine-ASPB31) (pi-H, 3.68 Å) (Triazole-ASPB31) (pi-H, 3.93 Å)
**8e**	−5.6498	1.7683	−8.3973	−5.7715	(Benzene-ASPB31) (pi-H, 3.94 Å) (Benzene-LYSB37) (pi-H, 3.55 Å)
Ligand	−3.6659	2.6939	−8.0864	−3.667	(O-GLNA209) (H-b, 3.11 Å) (O-LYSB37) (H-b, 3.20 Å) (O-GLYB41) (H-b, 3.16 Å)

**Table 6.** Results for molecular docking studies of compounds **3a, 4b** and **8e** versus reference in TLR-4 active site (PDB: 2Z63).

Compound	Docking score (s) Kcal/mol	RMSD	E score 1 (London dG) Kcal/mol	E score 2 (London dG) Kcal/mol	Binding interaction (ligand-receptor)
**3a**	−4.2663	1.5633	−8.003	−4.2663	(Benzene-GLU89) (pi-H, 3.75 Å) (Pyrimidine-PRO113) (pi-H, 3.82 Å) (Triazole-PRO113) (pi-H, 3.50 Å)
**4b**	−5.5773	1.4679	−8.1094	−4.575	(Triazole-GLU89) (pi-H, 3.98 Å) (Pyrrole-PRO113) (pi-H, 3.77 Å) (Benzene-PRO113) (pi-H, 3.83 Å) (Benzene-ASN137) (pi-H, 3.54 Å)
**8e**	−4.2089	1.5556	−7.8398	−4.2089	(Cl-GLU42) (H-b, 3.87 Å) (N Pyrimidine-GLU89) (H-b, 3.55 Å)
Ligand	−4.0874	1.9803	−8.1268	−3.7956	(O-ASN137) (H-b, 3.20 Å) (O-GLU89) (H-b, 3.07 Å)

**Figure 7.**
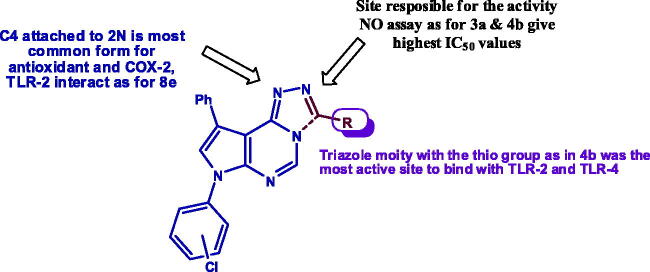
SAR for the active compounds with biological and docking results.

Using TLR-4 as the receptor protein, the reference ligand was found to interact in hydrophilic manner between its oxygen atoms and two amino acid residues (one hydrogen bond each) Glu 89 and Asn 137. Compound **4b** still shows the highest binding affinity with two additional binding interactions more than that of the reference ligand. It interacts with amino acid residues of Asn 137, Glu 89 and forms additional two hydrophobic interactions with with Pro 113 amino acid residue. Compound **3a** shows the second best binding affinity with three hydrophobic (aromatic interactions) with Glu 89 and Pro 113 amino acid residues. Finally, compound **8e** forms hydrophilic interactions with both Glu 42 and Glu 89 amino acid residues. These three compounds show the same interaction with Glu 89 as the ligand, and in line with the biological results indicated that the activities of both **3a** and **4b** is greater than **8e** in NO assay. To further understand the activities of the designed compounds, Figure 7 reveals the structure activity relationship (SAR) of the three active compounds.

**Figure 8.**
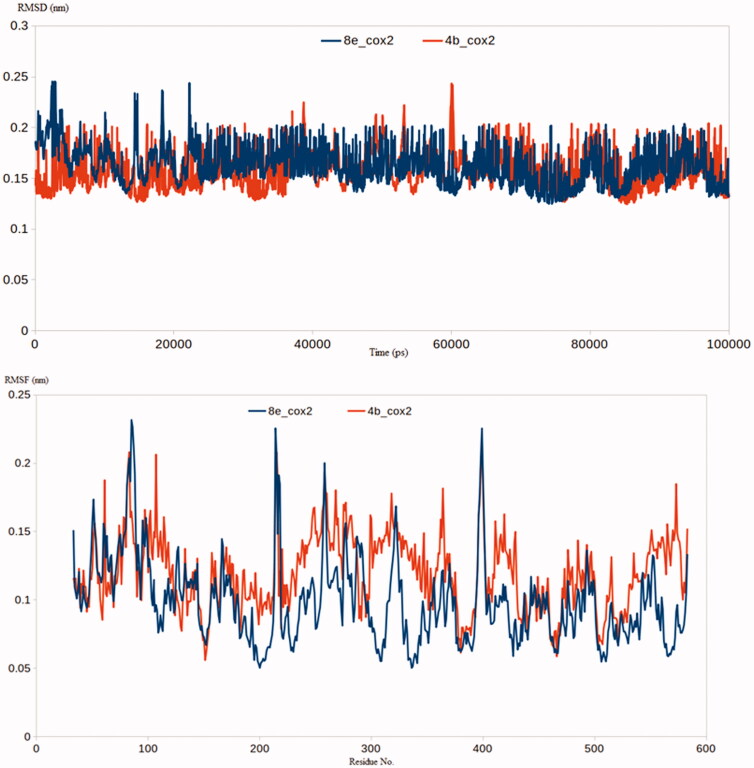
(a) RMSD analysis for the MD simulations of COX2-**4b** and COX2-**8e** complexes; (b) RMSF analysis for the MD simulations of COX2-**4b** and COX2-**8e** complexes.

The docking studies performed concluded that compounds **4b** and **8e** bind tightly to COX-2 and TLR-4 respectively. To further observe the stability of the complex as well as elaborate on the binding energy of interaction, molecular dynamic simulations was conducted for 100 ns using GROMACS 2.1.1 software. The MD simulation evaluation was conducted as follows: (1) ***RMSD and RMSF analysis*:** in the current work, further computational investigations were achieved through molecular dynamic simulations. Molecular dynamics (MD) simulation provides many valuable information and parameters to study the dynamicity of biological complexes. Amongst this information, MD could provide insights into precise estimation of the binding strength of a docked complex of a ligand and a target. Accordingly, the predicted binding co-ordinates retrieved from the docking of COX-2 and TLR-4 with **4b** and **8e** were moved forward to MD simulation. As demonstrated by Figure 8(a), the two proposed inhibitors had the privilege of forming a stable complex with COX-2 enzyme as indicated by their lower RMSD values. The COX2-**4b** and COX2-**8e** complexes had RMSD values of 0.2 nm and 0.19 nm, respectively. Similar results were obtained from the RMSF analysis where the residues of COX2-**4b** and COX2-**8e** complexes showed acceptable stabilities with an average RMSF of 0.18 and 0.17 nm, respectively Figure 8(b). The ability of compounds **4b** and **8e** to produce stable complexes as indicated by the low RMSD and RMSF values is a valid indicator on their inhibitory effect on COX-2 enzyme

**Figure 9.**
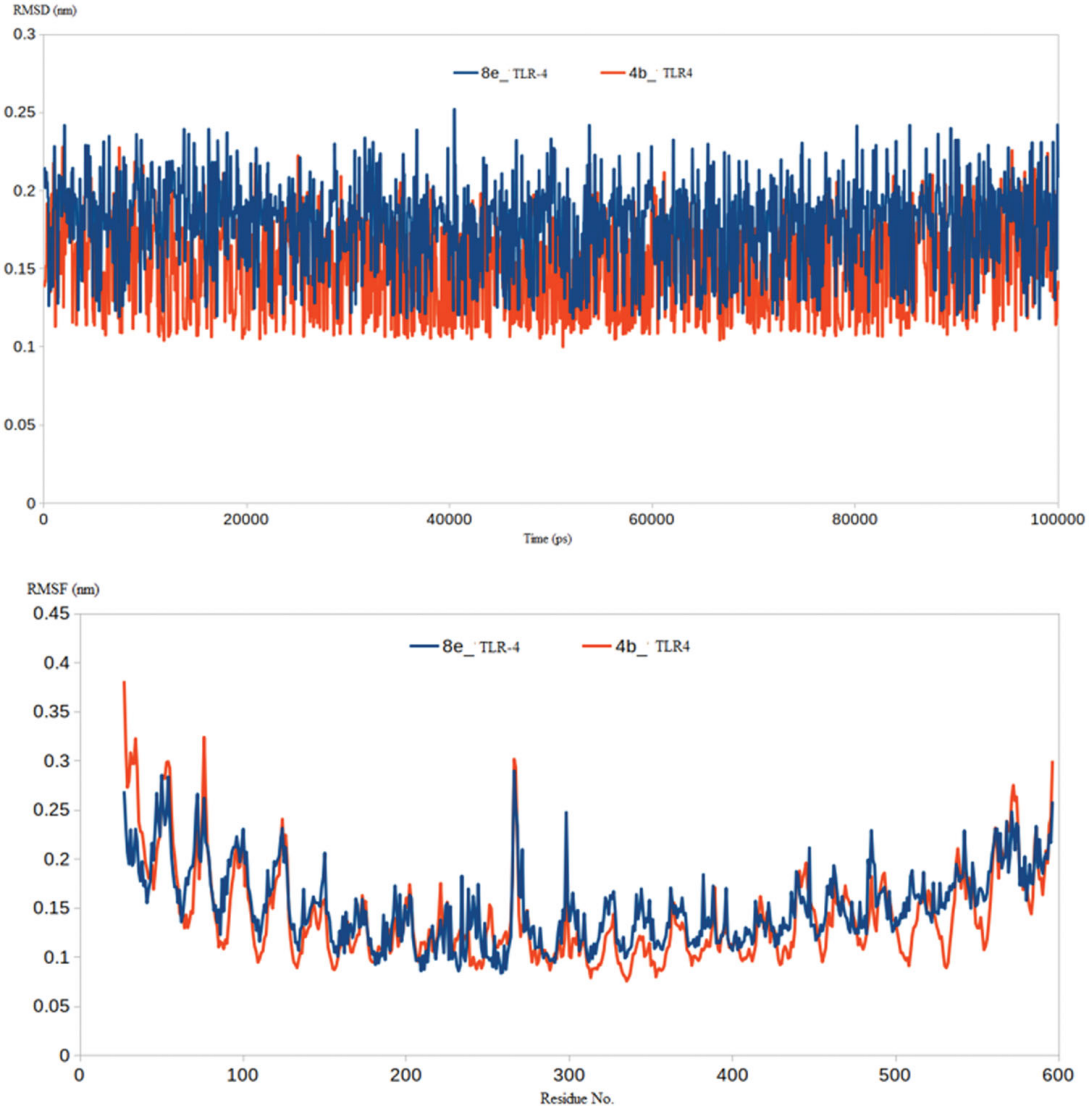
(a) RMSD analysis for the MD simulations of TLR-4-**4b** and TLR-4-**8e** complexes; (b) RMSF analysis for the MD simulations of TLR-4-**4b** and TLR-4-**8e** complexes.

Similar results were obtained from the molecular simulations of complexes of **4b** and **8e** with TLR-4. The TLR-4-**4b** and TLR-4-**8e** complexes had RMSD values of 0.18 nm and 0.22 nm, respectively Figure 9(a). Besides, most of the residues of TLR-4-**4b** and TLR-4-**8e** complexes reached an average RMSF of 0.19 and 0.21 nm, respectively Figure 9(b). In conclusion, the RMSD and RMSF analysis of the formed complexes between **4b** and **8e** with COX-2 and TLR-4 showed favourable stability for both the compounds and emphasised the results from the experimental assays. (2) ***Binding Free Energy Calculations using MM-PBSA approach*:** attempting to further endorse the binding strength between the COX-2 enzyme and TLR-4 with the newly developed compounds **4b** and **8e**, the *g_mmpbsa* package was brought in action to compute the binding free energies between the two targets and the proposed molecules **4b** and **8e**. The generated trajectories from the production stage were used to calculate all the forms of binding free energy. These energy types include electrostatic energy, van der Waal energy, polar solvation energy and SASA energy. All the previous types of energy were calculated for the four complexes containing COX-2 and TLR-4 bound to **4b** or **8e** (Table 7).

**Table 7.** The binding free energies of **4b** and **8e** in complex with COX-2 and TLR-4.

Complex	ΔE_binding (kJ/mol)_	ΔE_Electrostatic (kJ/mol)_	ΔE*_Vander Waal_*_(kJ/mol)_	ΔE_polar solvation (kJ/mol)_	SASA _(kJ/mol)_
**4b**-COX-2	−87 ± 2.3	−57.1 ± 2.2	−73.1 ± 2.3	56.9 ± 1.3	−13.7 ± 0.1
**8e**-COX-2	−89.1 ± 2.0	−62.5 ± 1.9	−69.9 ± 2.0	55.8 ± 1.2	−12.5 ± 0.2
**4b**-TLR-4	−106.2 ± 1.8	−65.6 ± 1.8	−77.7 ± 1.9	54.4 ± 1.5	−17.3 ± 0.2
**8e**-TLR-4	−81.9 ± 1.5	−55.1 ± 1.5	−59.7 ± 1.6	48.7 ± 1.4	−15.8 ± 0.1

Interestingly as shown in Table 7, the calculated binding free energy for the two small molecules were favourable in which compound **4b** achieved binding free energies of −87 ± 2.3 and −106.2 ± 1.8 (kJ/mol) with COX-2 and TLR-4, respectively. On the other hand, compound **8e** achieved binding free energies of −89.1 ± 2.0and −81.9 ± 1.5 (kJ/mol) with COX-2 and TLR-4, respectively These results augmented all the *in-silico* calculations giving credit to the predicted binding mode of both **4b** and **8e** within COX-2 and TLR-4 binding sites.

## Conclusions

In this study, we have presented the design, synthesis and biological evaluation of novel arylidineaminopyrrolopyrimidine, and their fused form triazolo-pyrrolopyrimidine, that can act as antioxidant and anti-inflammatory promising agents utilising LPS-induced macrophages (RAW264.7) cells. The present study report that pyrrolopyrimidines (**3a, 4b** and **8e**) manage to fit in new Toll 2 and 4 receptors in exceptionally great manners supported by molecular modelling and simulations compared to well-known ligand. In addition, we reported their mechanism action as anti-COX2 agents, as conceivable proposed novel pathway for their anti-inflammatory aside with anti-oxidant activities.
